# Supplementary material for: Phenotypic changes of HER2-positive breast cancer during and after dual HER2 blockade
Source: Nat Commun. 2020 Jan 20;11:385. doi: 10.1038/s41467-019-14111-3 (PMC6971277; doi:10.1038/s41467-019-14111-3)
Supplement: Supplementary file 2 — Supplementary Information [file 41467_2019_14111_MOESM2_ESM.pdf]

Supplementary Information

Brasó-Maristany et al.

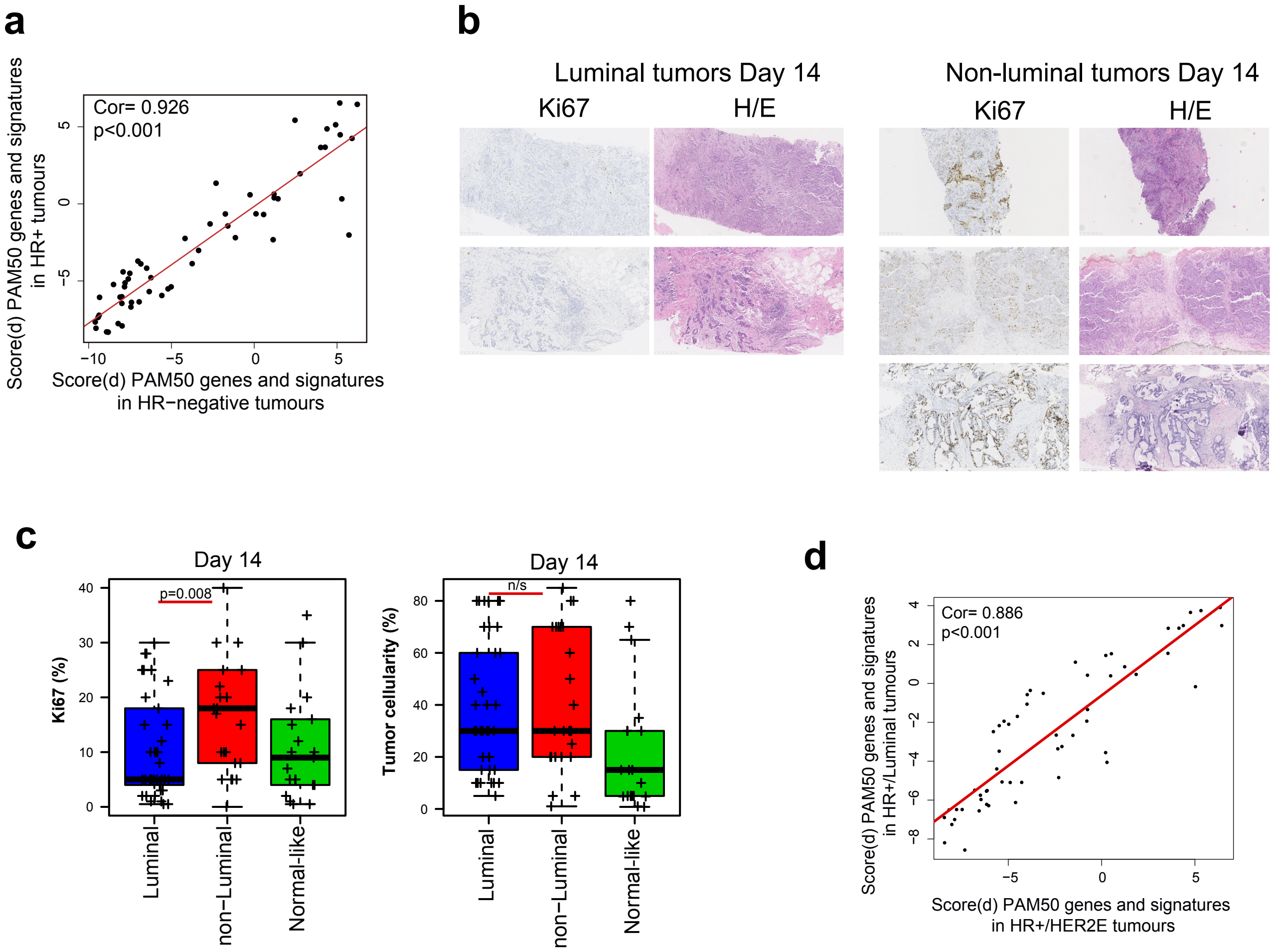

Supplementary Figure 1. Molecular changes in HER2-E tumors upon dual HER2-blockade. **(a)** Pearson correlation between differential PAM50 gene and signatures expression (Score [d]) in HER2+/HR+/HER2-E and HER2+/HR-negative/HER2-E tumors after 2 weeks of treatment with trastuzumab plus lapatinib. **(b)** Representative images of Ki67 and H/E stainings in Luminal and non-Luminal tumors of the PAMELA trial at day 14 **(c)** Ki67 (%) and tumor cellularity (%) at day 14 across molecular subtypes. P-values were determined by two-tailed unpaired t-tests. **(d)** Pearson correlation between differential PAM50 gene and signatures expression (Score [d]) in HER2+/HR+/HER2-E and HER2+/HR+/Luminal tumors after 2 weeks of treatment with trastuzumab plus lapatinib. Source data are provided as a Source Data file.

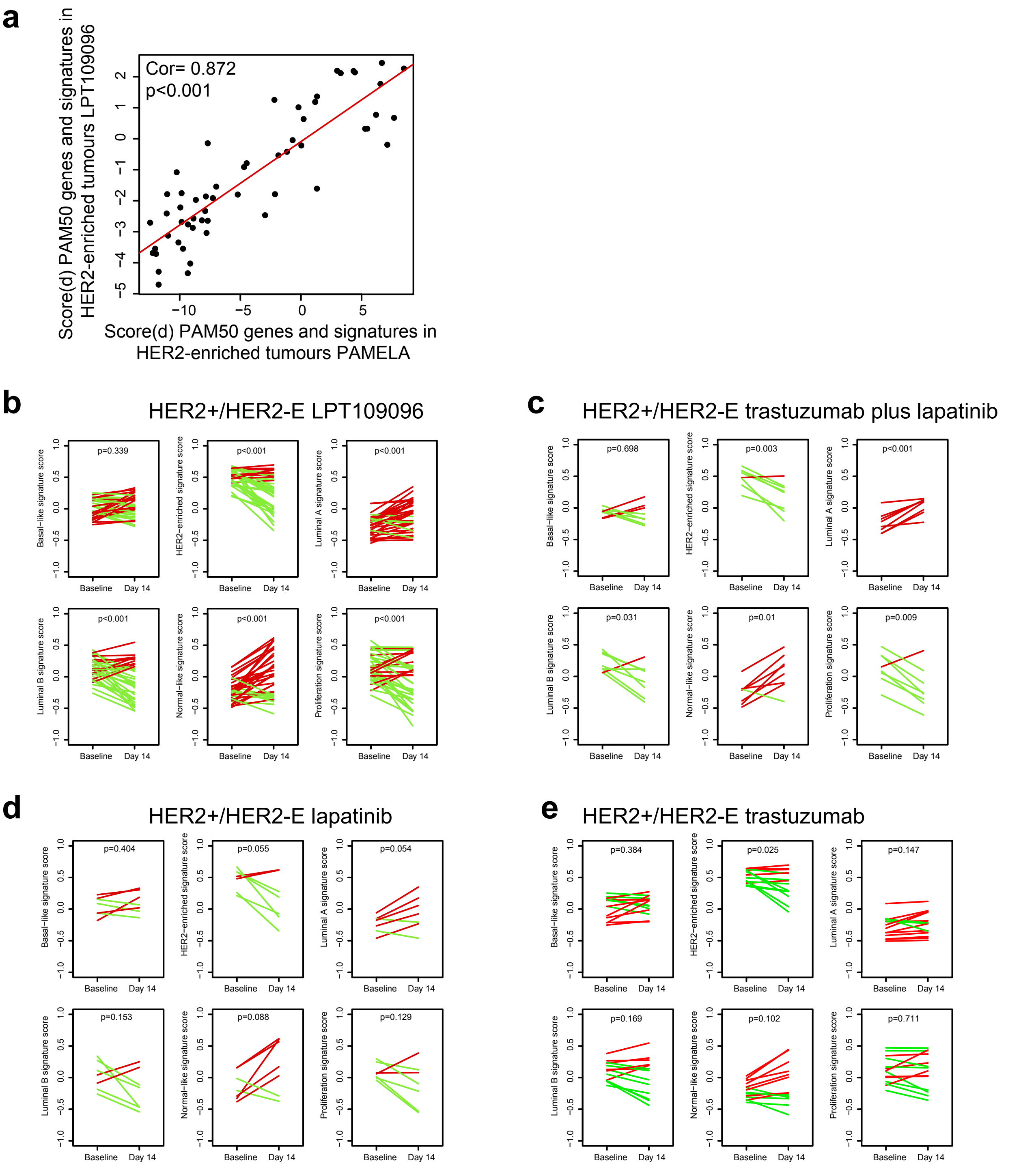

Supplementary Figure 2. HER2 blockade led to a more luminal-like phenotype in HER2-E tumors of the LPT109096 trial. (a) Pearson correlation between differential PAM50 gene and signatures expression (Score [d]) in HER2-E tumors of the PAMELA study and HER2-E tumors of the LPT109096 study. **(b)** PAM50 signature expression changes between baseline and day 14 tumors treated with lapatinib, trastuzumab or the combination of both. Each line represents a tumor sample. P-values were determined by two-tailed paired t-tests. **(c)** PAM50 signature expression changes between baseline and day 14 tumors treated with lapatinib plus trastuzumab. **(d)** PAM50 signature expression changes between baseline and day 14 tumors treated with lapatinib. **(e)** PAM50 signature expression changes between baseline and day 14 tumors treated with trastuzumab. Each line represents a tumor sample. P-values were determined by two-tailed paired t-tests.

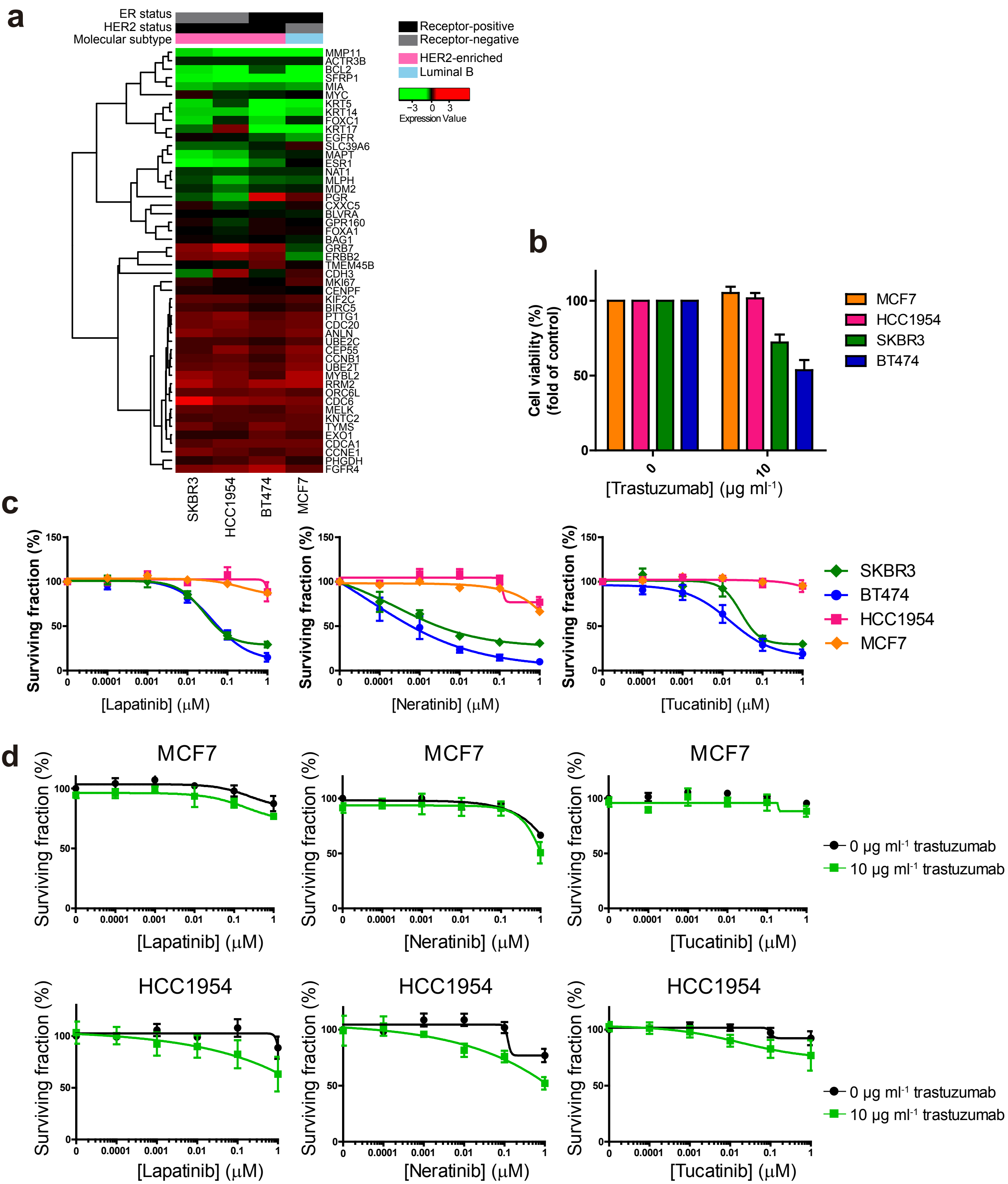

Supplementary Figure 3. Characterization of breast cancer cell lines. (a) Unsupervised hierarchical clustering of BT474, SKBR3, HCC1954 and MCF7 cell lines. The heatmap shows high (red) to low (green) expression of mRNAs in each sample. The molecular subtype call and HER2 and HR expression of each sample are shown. **(b)** Cell viability (%) of BT474 SKBR3, HCC1954 and MCF7 cells upon treatment with 10 µg ml^{-1} trastuzumab or **(c)** increasing concentrations of lapatinib, neratinib or tucatinib for 72 hours. **(d)** Cell viability (%) of HCC1954 and MCF7 cells upon treatment with increasing concentrations of the TKI lapatinib, neratinib or tucatinib as monotherapy or in combination with 10 µg ml^{-1} trastuzumab for 72 hours. Data points in **(b), (c)** and **(d)** represent the mean; error bars represent the standard error of the mean of 3 independent experiments. Source data are provided as a Source Data file.

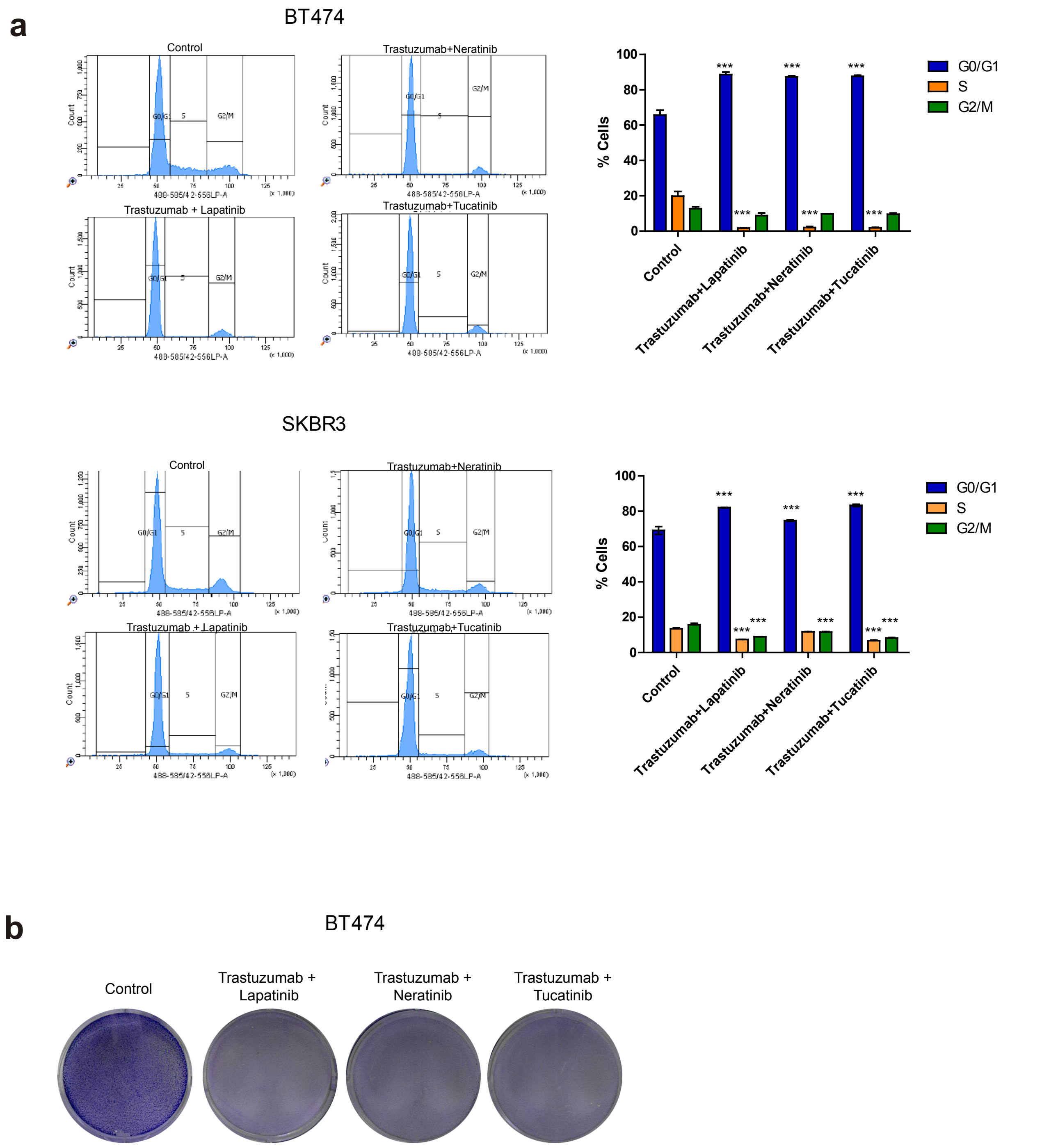

Supplementary Figure 4. Effects of anti-HER2 treatments on cell cycle and clonogenic potential. **(a)** Cell cycle analysis of BT474 and SKBR3 control an treated with 10 µg ml^{-1} trastuzumab plus TKI (10 nM lapatinib, 2 nM neratinib or 10 nM tucatinib) or DMSO control and quantification of % of cells in each cell cycle phase. Column bars represent the mean of 3 independent experiments; error bars represent the standard error of the mean. P-values (***: $p<0.001$) were determined by two-way ANOVAs. **(b)** Representative images of BT474 treated with with DMSO control or combinations of 10 µg ml^{-1} trastuzumab and TKI (10 nM lapatinib, 2 nM neratinib or 10 nM tucatinib) and stained with 0.5% crystal violet solution after 10 days. Source data are provided as a Source Data file.

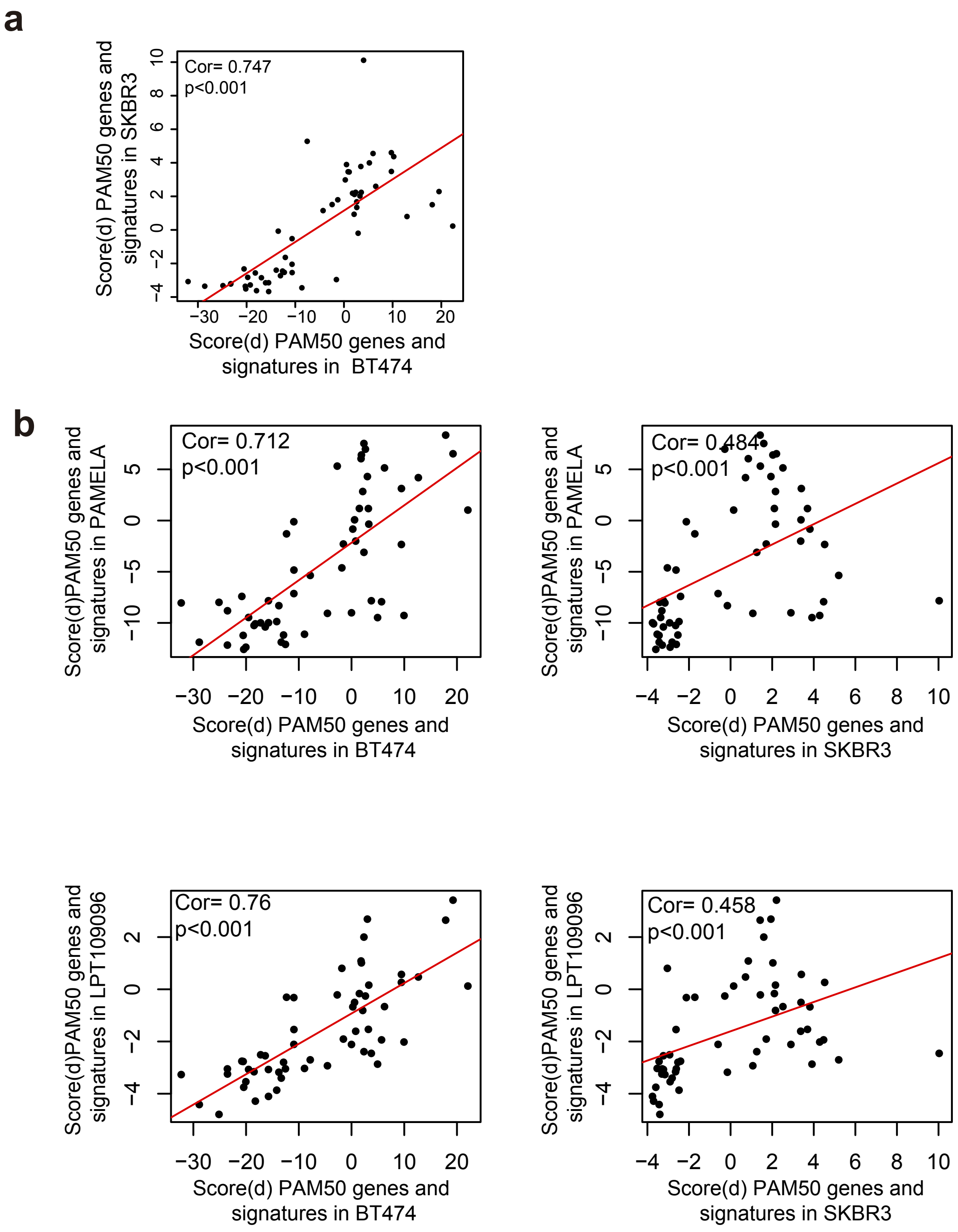

Supplementary Figure 5. Gene and signatures changes in HER2+/HR+/HER2-E and HER2+/HR-negative/HER2-E cell lines and tumors upon dual HER2-blockade. (a) Pearson correlation between differential PAM50 gene and signatures expression (Score [d]) in BT474 and SKBR3 cells. **(b)** Pearson correlations between differential PAM50 gene and signatures expression (Score [d]) in TKI and trastuzumab-treated breast cancer cell lines and HER2-E tumors of the PAMELA and LPT109096 trials.

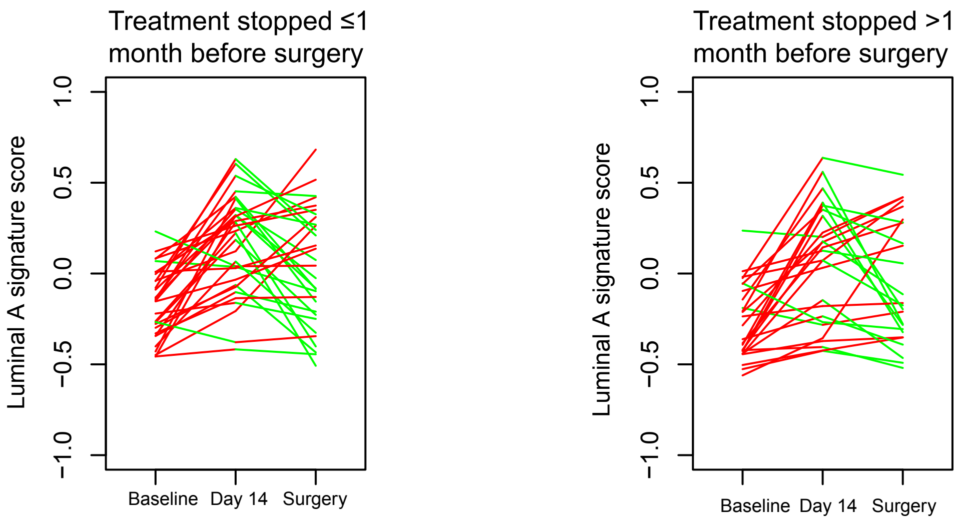

Supplementary Figure 6. Biological changes after dual HER2 blockade. Luminal A signature expression changes between baseline, day 14 and surgery in residual tumors, separated by time from end of treatment to surgery. Source data are provided as a Source Data file.

Supplementary Table 1. Gene expression profiles between day 14 and baseline in tumors of the PAMELA trial. Lists of differentially expressed genes between day 14 and baseline in tumors HER2+/HR+/HER2-E and tumors HER2+/HR-negative/HER2-E determined by SAM analysis.

SAM results HER2+/HR+/HER2E		
PAM50 gene or signature	Score (d)	q-value (% FDR)
SFRP1	6.46970618	0
Normal	6.3872984	0
EGFR	5.35663022	0
BCL2	5.06052018	0
FOXC1	4.79417774	0
LumA	4.41129528	0
KRT14	4.17549989	0
KRT5	3.60365709	0
MIA	3.58795374	0
BAG1	1.88401491	0.33673772
PHGDH	1.26924536	1.8978564
KRT17	0.55727256	10.0937572
Basal	0.51615725	11.2848065
MYC	0.32153393	13.5441796
MDM2	0.25715744	14.9731305
PGR	0.24875259	14.9731305
ANLN	-8.392227	0
MYBL2	-8.3792142	0
CCNB1	-8.1403335	0
MKI67	-7.9912978	0
BIRC5	-7.8405124	0
UBE2C	-7.7368716	0
CENPF	-7.4374211	0
RRM2	-7.2959853	0
KIF2C	-6.7642229	0
CEP55	-6.5278776	0
KNTC2	-6.4604716	0
CDC20	-6.4274859	0
UBE2T	-6.1347616	0
EXO1	-6.124016	0
ORC6L	-6.0978949	0
TYMS	-6.0167558	0
Her2	-5.765923	0
Proliferation.	-5.5845645	0
LumB	-5.4551036	0
ERBB2	-5.4505663	0
CDCA1	-5.3032377	0
GRB7	-5.1956307	0
FOXA1	-4.9427805	0
PTTG1	-4.8639359	0
CDC6	-4.5760108	0
FGFR4	-4.4812287	0
MELK	-4.2474824	0
MLPH	-3.9638204	0
GPR160	-3.9515314	0
TMEM45B	-3.7870791	0
MMP11	-3.0931685	0
NAT1	-2.3986462	0
CCNE1	-2.3133168	0
MAPT	-2.2670435	0
ESR1	-2.0921499	0.10616361
SLC39A6	-1.5042711	0.64653641
CDH3	-1.3705641	1.17909376
CXXC5	-0.7606059	6.78580491
ACTR3B	-0.7238199	6.78580491
BLVRA	-0.7188276	6.78580491

SAM results HER2+/HR-/HER2E		
PAM50 gene or signature	Score (d)	q-value (% FDR)
Normal	6.15800003	0
KRT14	5.84216192	0
ESR1	5.65647281	0
PGR	5.22656919	0
LumA	5.12669179	0
SFRP1	5.10093245	0
BCL2	4.85173226	0
FOXC1	4.32964763	0
KRT5	4.21822733	0
MIA	3.95104105	0
BAG1	2.70717314	0
EGFR	2.39800331	0.04977261
MDM2	1.3710356	2.19966358
MYC	1.13949548	3.58489853
KRT17	1.12936489	3.58489853
NAT1	1.07697888	3.58489853
CXXC5	0.50455003	11.4058563
BLVRA	0.04393207	14.4021793
UBE2C	-9.6658332	0
CCNB1	-9.6213003	0
CENPF	-9.4923592	0
RRM2	-9.4309033	0
UBE2T	-9.4026078	0
MYBL2	-8.9779165	0
ANLN	-8.882521	0
CDCA1	-8.5615767	0
BIRC5	-8.2813934	0
EXO1	-8.1651828	0
ORC6L	-8.057896	0
CEP55	-8.0559901	0
MKI67	-8.0426157	0
FGFR4	-7.9837552	0
ERBB2	-7.8954499	0
GRB7	-7.8623169	0
FOXA1	-7.6709285	0
CDC6	-7.5776642	0
KIF2C	-7.5096191	0
KNTC2	-7.476962	0
TMEM45B	-7.088461	0
CDC20	-7.0125249	0
MLPH	-6.90992	0
MELK	-6.5593125	0
Her2	-6.4054409	0
PTTG1	-6.3005764	0
TYMS	-5.6443705	0
Proliferation.	-5.2700002	0
LumB	-5.0820503	0
CCNE1	-4.2419873	0
GPR160	-3.811707	0
MMP11	-3.4240648	0
CDH3	-2.7305932	0
PHGDH	-2.3708076	0
ACTR3B	-1.8091845	0.18725818
SLC39A6	-1.6584347	0.49329089
MAPT	-1.2022239	1.52018129
Basal	-0.3165352	10.6355494

n (%) genes which expression significantly changed at day 14		
PAM50 genes and signatures	HER2+/HR+/HER2-E	HER2+/HR-/HER2-E
significantly upregulated	11 (19.6%)	16 (28.6%)
significantly downregulated	37 (66.1%)	37 (66.1%)
significantly changed	48 (85.7%)	53 (94.7%)

Supplementary Table 2. Gene expression profiles between day 14 and baseline in tumors of the LPT109096 trial. Lists of differentially expressed genes between day 14 and baseline in tumors HER2+/HER2-E determined by SAM analysis.

SAM results HER2+/HER2E		
PAM50 gene or signature	Score (d)	q-value (% FDR)
LumA	2.41838424	0.364256025
Normal	2.236725985	0.689984971
ESR1	2.165060269	0.689984971
PGR	2.156161758	0.689984971
EGFR	2.112983956	1.116242352
BAG1	2.087011993	1.116242352
BCL2	1.741287709	2.3084056
MDM2	1.335338364	6.211924392
MAPT	1.225459931	8.045340075
MYC	1.158344635	8.045340075
BLVRA	0.985612937	11.9013993
FOXC1	0.743408576	17.53002912
SFRP1	0.64600633	19.56687468
CXXC5	0.606938823	21.45793777
KRT5	0.296018843	28.84689165
MIA	0.294162017	28.84689165
RRM2	-4.734293694	0
ERBB2	-4.367722434	0
ANLN	-4.317934529	0
GRB7	-4.054252844	0
CENPF	-3.743732209	0
UBE2C	-3.715674287	0
KNTC2	-3.576202236	0
MYBL2	-3.574679304	0
EXO1	-3.374244041	0
MKI67	-3.154297258	0
MLPH	-3.067889774	0
FGFR4	-2.902270025	0
CDC20	-2.792382984	0
CCNB1	-2.736870423	0
KIF2C	-2.710805965	0
TMEM45B	-2.674148145	0
Her2	-2.659903209	0
FOXA1	-2.600037815	0
CDH3	-2.494727115	0
BIRC5	-2.439357783	0
TYMS	-2.357887728	0
ORC6L	-2.245064323	0.227083661
CDC6	-2.000992396	0.364256025
Proliferation.Score	-1.942576112	0.364256025
PTTG1	-1.88976293	0.689984971
GPR160	-1.827838843	0.689984971
SLC39A6	-1.816706527	0.689984971
UBE2T	-1.816652365	0.689984971
CDCA1	-1.78452686	1.116242352
KRT17	-1.637900104	1.611761767
LumB	-1.57289301	2.3084056
CEP55	-1.109609116	8.045340075
CCNE1	-0.941149962	11.9013993
MMP11	-0.818230781	15.58135075
ACTR3B	-0.564088278	22.93979819
PHGDH	-0.44914754	25.99564997
Basal	-0.244066193	32.09534609
KRT14	-0.221571796	32.09534609
MELK	-0.174463958	33.01686895
NAT1	-0.073726747	34.14101136

changed at day 14	
PAM50 genes and signatures	HER2+/HER2-E
significantly upregulated	11 (19.6%)
significantly downregulated	37 (66.1%)
significantly changed	48 (85.7%)

Supplementary Table 3. Gene expression profiles between anti-HER2-treated and control cell lines. Lists of differentially expressed genes between treated and untreated BT474 and SKBR3 determined by SAM analysis.

SAM results BT474			SAM results SKBR3		
PAM50 gene or signature	Score (d)	q-value (% FDR)	PAM50 gene or signature	Score (d)	q-value (% FDR)
MYC	22.3093313	0	TMEM45B	10.0915537	0
LumA	19.4924567	0	GPR160	5.26141284	0
Normal	18.0966483	0	MAPT	4.58506588	0
PGR	12.9120696	0	MLPH	4.5349134	0
GRB7	10.191529	0	GRB7	4.3477998	0
BAG1	9.70542779	0	ERBB2	3.97622206	0
MAPT	9.69600973	0	NAT1	3.87621467	0
MIA	6.49544207	0	KRT17	3.76005902	0
MLPH	5.93731448	0	BAG1	3.46366279	0
ERBB2	5.18130394	0	CXXC5	3.44629861	0
TMEM45B	3.99625975	0	ACTR3B	3.43351463	0
BLVRA	3.51777631	0	FOXA1	2.96195992	0
KRT17	3.4346435	0	MIA	2.57853407	0
EGFR	3.24853725	0	LumA	2.26626769	0
KRT14	2.87140641	0	ESR1	2.22124563	0
CDH3	2.59820706	0	BLVRA	2.21929742	0
SFRP1	2.59077176	0	MDM2	2.16674336	0
ESR1	2.36648054	0	BCL2	2.09600592	0
BCL2	2.11642018	0	EGFR	2.00173608	0
FOXC1	2.04696068	0	SLC39A6	1.77622477	0.31055901
MDM2	1.72646494	0	SFRP1	1.65735565	0.31055901
ACTR3B	1.03365261	0.58309038	KRT5	1.49284381	0.58309038
CXXC5	0.81751484	0.85714286	Normal	1.48261777	0.58309038
NAT1	0.48534011	2.38095238	CDH3	1.32020875	0.58309038
FOXA1	0.23442552	2.38095238	FGFR4	1.13126342	0.85714286
TYMS	-32.061148	0	FOXC1	0.91334586	2.1978022
RRM2	-28.634145	0	PGR	0.77909601	2.1978022
PTTG1	-24.892848	0	MYC	0.20920028	11.9480519
MYBL2	-23.299509	0	KIF2C	-3.6938286	0
CDC6	-23.28624	0	ORC6L	-3.6409611	0
Proliferation.	-20.562718	0	CCNB1	-3.5339459	0
BIRC5	-20.309112	0	MKI67	-3.4688951	0
CCNB1	-20.206106	0	BIRC5	-3.3777159	0
UBE2C	-19.802115	0	RRM2	-3.3736596	0
CDC20	-19.282807	0	PTTG1	-3.3409174	0
EXO1	-18.248942	0	CDC20	-3.3005879	0
ORC6L	-17.997795	0	CDC6	-3.2433842	0
CDCA1	-16.992776	0	MYBL2	-3.2245953	0
CEP55	-16.130011	0	CEP55	-3.1708805	0
MELK	-15.51551	0	MELK	-3.1673059	0
KIF2C	-15.503251	0	TYMS	-3.0971865	0
KNTC2	-13.946895	0	MMP11	-2.9792742	0
Her2	-13.538759	0	CDCA1	-2.8695331	0
ANLN	-13.083033	0	UBE2C	-2.8459982	0
UBE2T	-12.651167	0	ANLN	-2.7497298	0
CENPF	-12.291133	0	EXO1	-2.583306	0
PHGDH	-12.085984	0	CCNE1	-2.5630939	0
Basal	-10.703039	0	CENPF	-2.5489344	0
LumB	-10.697143	0	UBE2T	-2.4714759	0
CCNE1	-10.684727	0	KNTC2	-2.4181577	0
MKI67	-8.6796733	0	Proliferation.	-2.3439252	0
GPR160	-7.5904084	0	Basal	-2.0669437	0
FGFR4	-4.3265546	0	PHGDH	-1.6582951	0.31746032
KRT5	-2.470231	0	LumB	-0.5401174	5.92991914
MMP11	-1.5908671	0	KRT14	-0.2170521	11.9480519
SLC39A6	-1.3072353	0	Her2	-0.093242	12.5

n (%) genes which expression significantly changed at 72 h		
PAM50 genes and signatures	BT474	SKBR3
significantly upregulated	25 (44.6%)	27 (48.2%)
significantly downregulated	31 (55.4%)	25 (44.6%)
significantly changed	56 (100%)	52 (92.8%)

Supplementary Table 4. Gene expression profiles between surgery and day 14 in residual tumors of the PAMELA trial. Lists of differentially expressed genes between surgery and day 14 in residual HER2+/HER2-E tumors determined by SAM analysis.

SAM results day 14 vs residual tumor		
PAM50 gene or signature	Score (d)	q-value (% FDR)
MYC	5.9969978	0
KRT17	4.16496599	0
KRT5	3.62457159	0
UBE2T	3.2318067	0
MIA	2.99023234	0
KRT14	2.98090971	0
EXO1	2.79177628	0
FOXC1	2.78401243	0
PHGDH	2.74123057	0
CDCA1	2.68866523	0
MAPT	2.33025674	0
SFRP1	2.27962982	0
TYMS	2.20944238	0
BLVRA	2.17584252	0
CEP55	1.95103282	0
PGR	1.82393423	0
RRM2	1.81633076	0
BIRC5	1.7594765	0
CDC20	1.72158454	0
UBE2C	1.66039707	0
TMEM45B	1.52234215	0.060299
BCL2	1.50609152	0.060299
MKI67	1.4726498	0.060299
MYBL2	1.42804655	0.060299
ESR1	1.42751414	0.060299
MLPH	1.3978971	0.060299
FOXA1	1.35480731	0.060299
CCNB1	1.33468156	0.060299
KNTC2	1.33205903	0.060299
CDC6	1.28713127	0.10611059
EGFR	1.28514889	0.10611059
MELK	1.17950362	0.10611059
FGFR4	1.12902328	0.10611059
GRB7	1.03156326	0.37033496
CCNE1	0.96221905	0.37033496
CDH3	0.83782518	0.61169632
ANLN	0.81149944	0.61169632
Basal	0.73394152	0.61169632
KIF2C	0.53338263	0.61169632
Normal	0.47183479	0.61169632
CENPF	0.40750995	0.61169632
Proliferation.	0.39116135	0.61169632
BAG1	0.37724082	0.61169632
CXXC5	0.3684763	0.61169632
PTTG1	0.34998073	0.61169632
ORC6L	0.31363867	0.61169632
MMP11	-4.3451389	0
MDM2	-2.8387937	0
GPR160	-1.2155317	0.76999619
ACTR3B	-1.1722067	0.76999619
SLC39A6	-0.700598	2.19902495
LumA	-0.5086705	3.94816837
Her2	-0.4185061	4.62754837
LumB	-0.3504179	5.20758105
NAT1	-0.3113614	5.95026643
ERBB2	-0.1898128	7.06591756

n (%) genes which expression significantly changed	
PAM50 genes and signatures	HER2+/HER2-E
significantly upregulated	46 (82.1%)
significantly downregulated	6 (10.7%)
significantly changed	52 (92.8%)

Supplementary Table 5. Gene expression profiles after anti-HER2 treatment discontinuation in breast cancer cell lines. Lists of differentially expressed genes between cells after treatment discontinuation and treated with anti-HER2 therapy for 72 hours determined by SAM analysis.

SAM results BT474		
PAM50 gene or signature	Score (d)	q-value (% FDR)
UBE2T	5.64771526	0
RRM2	5.24025011	0
TYMS	4.24155798	0
CDC6	3.94945638	0
FGFR4	3.93932464	0
MYBL2	3.67725006	0
PTTG1	3.56187941	0
CDC20	3.45738846	0
MELK	3.34569468	0
EXO1	3.30394814	0
BIRC5	3.27321788	0
PHGDH	3.26564704	0
MKI67	3.25073895	0
ANLN	3.1825882	0
CEP55	3.15995457	0
KIF2C	3.1153988	0
ORC6L	2.88117683	0.74336283
Proliferation.	2.84342528	0.74336283
CDCA1	2.7689423	0.74336283
CCNB1	2.68177325	0.74336283
KNTC2	2.63064025	0.74336283
GPR160	2.50463998	0.74336283
Her2	2.45978513	0.74336283
BCL2	2.45378207	0.74336283
CENPF	2.42081411	0.74336283
UBE2C	2.40523987	0.74336283
LumB	2.06449157	1.85840708
Basal	1.76540029	2.73295159
KRT5	1.71286567	2.73295159
MMP11	1.67434709	2.73295159
ACTR3B	1.01839603	11.192679
SLC39A6	0.62333832	20.1327434
PGR	0.43927746	23.7569565
NAT1	0.2740484	26.8241777
ESR1	0.2545274	26.8241777
FOXA1	0.22928433	26.8241777
GRB7	-8.952422	0
ERBB2	-3.0556566	0.86437539
Normal	-2.9893113	0.86437539
LumA	-2.8594964	0.86437539
EGFR	-2.5975391	0.86437539
MIA	-2.5708598	0.99557522
CDH3	-2.509935	0.99557522
KRT14	-1.7950501	2.73295159
BLVRA	-1.6474238	2.73295159
TMEM45B	-1.6443933	2.73295159
BAG1	-1.3957195	3.76704138
MLPH	-1.3093719	4.46017699
MDM2	-1.1663668	5.24166099
SFRP1	-0.9930443	5.24166099
MYC	-0.7388819	11.192679
FOXC1	-0.5256456	15.4208247
MAPT	-0.5018061	15.4208247
CXXC5	-0.3430367	15.4208247
KRT17	-0.1465987	15.4208247
CCNE1	-0.0076394	15.4208247

SAM results SKBR3		
PAM50 gene or signature	Score (d)	q-value (% FDR)
BIRC5	3.62129373	0
PTTG1	3.48268016	0
ORC6L	3.41702299	0
PHGDH	3.25301632	0
CDC20	3.22496063	0
UBE2T	3.06679189	0
ANLN	3.06503655	0
CDCA1	2.88292653	0
CCNB1	2.88220608	0
TYMS	2.87625166	0
CEP55	2.86025669	0
EXO1	2.82205515	0
CENPF	2.73825151	0
CCNE1	2.60727219	0
CDC6	2.57438742	0
MELK	2.50763625	0
RRM2	2.47517231	0
KNTC2	2.40691848	0
MYBL2	2.35049619	0
KIF2C	2.33974306	0
Basal	1.93332396	0.46082949
UBE2C	1.93086974	0.46082949
Proliferation.	1.89036537	0.46082949
SLC39A6	1.75907523	0.46082949
ACTR3B	1.15951435	4.34782609
MYC	1.0266555	4.34782609
LumB	1.0146316	4.34782609
ESR1	0.92097902	4.34782609
MKI67	0.90548013	4.34782609
MMP11	0.76955818	4.34782609
CXXC5	0.48956207	4.34782609
EGFR	0.28047244	4.34782609
BLVRA	0.21647794	4.34782609
BCL2	0.03462159	4.34782609
KRT14	0.02832246	4.34782609
MDM2	-5.8174899	0
FGFR4	-4.4747558	0
MLPH	-4.1277234	0
FOXA1	-4.0080666	0
TMEM45B	-3.6545581	0
GRB7	-3.5113559	0
KRT17	-2.7378484	0
MIA	-2.2003798	0.89285714
LumA	-2.0920206	0.89285714
NAT1	-2.0350607	0.89285714
ERBB2	-2.0012702	0.89285714
MAPT	-1.887994	0.89285714
BAG1	-1.7256374	0.89285714
SFRP1	-1.6069372	0.89285714
Normal	-1.5736336	4.34782609
GPR160	-1.4821297	4.34782609
KRT5	-1.4577348	4.34782609
FOXC1	-1.1323695	4.86322188
CDH3	-0.4231845	4.86322188
PGR	-0.2600519	4.86322188
Her2	-0.1188024	4.86322188

n (%) genes which expression significantly changed upon discontinuation		
PAM50 genes and signatures	BT474	SKBR3
significantly upregulated	30 (53.6%)	35 (62.5%)
significantly downregulated	12 (35.7%)	21 (37.5%)
significantly changed	56 (75%)	56 (100%)

Supplementary Table 6. Gene expression profiles in BT474-L^{R}T^{R} and BT474-Tu^{R}T^{R}. Lists of differentially expressed genes between BT474-L^{R}T^{R} and parental BT474 cells and between BT474-Tu^{R}T^{R} and parental BT474 cells determined by SAM analysis.

SAM results BT474 vs BT474-LRTR		
**PAM50 genes**	**Score (d)**	**q-value (% FDR)**
FGFR4	12.7323407	0
FOXA1	9.11196718	0
GRB7	6.45458	0
ERBB2	5.91971466	0
MLPH	5.49257597	0
KRT5	4.58430964	0
EGFR	4.3881213	0
MDM2	3.90096637	0
MAPT	3.77590049	0
SFRP1	3.59380934	0
BAG1	2.39454049	1.14942529
GPR160	1.9489275	1.64576803
BLVRA	1.87658348	1.64576803
MIA	1.57990066	2.09895053
CXXC5	1.50736934	2.09895053
NAT1	1.22941738	2.51436782
PGR	1.08497893	2.51436782
ESR1	0.81518396	2.51436782
ACTR3B	0.2820106	2.51436782
MYC	0.09596955	2.51436782
KRT14	-0.0777179	1.17746005
KRT17	-0.1676905	1.17746005
FOXC1	-0.3858942	1.17746005
SLC39A6	-0.4864237	1.17746005
TMEM45B	-0.4948573	1.17746005
MMP11	-1.0146634	1.17746005
BCL2	-1.1802496	1.17746005
PHGDH	-1.2890688	1.17746005
CDH3	-1.8645395	0.60344828
MKI67	-3.7996034	0
CCNE1	-4.6235586	0
UBE2T	-5.0562345	0
UBE2C	-6.4896599	0
KNTC2	-7.7897848	0
CDC6	-10.018136	0
KIF2C	-10.976417	0
PTTG1	-11.189744	0
CDC20	-12.55016	0
ORC6L	-14.127434	0
CENPF	-14.251581	0
MELK	-14.81298	0
RRM2	-15.219372	0
BIRC5	-16.038778	0
ANLN	-16.857195	0
EXO1	-18.261239	0
CEP55	-19.811407	0
TYMS	-21.079682	0
CDCA1	-22.710382	0
MYBL2	-25.104831	0
CCNB1	-26.321783	0

SAM results BT474 vs BT474-TuRTR		
**PAM50 genes**	**Score (d)**	**q-value (% FDR)**
FGFR4	7.03476586	0
KRT5	5.53855821	0
KRT14	3.9474193	0
SFRP1	3.9474193	0
ERBB2	3.26755369	0
MAPT	3.10027698	0
EGFR	3.02649972	0
MDM2	3.01789419	0
FOXA1	2.16563796	1.03448276
MLPH	1.56457514	7.21732157
GRB7	1.43635636	7.21732157
FOXC1	1.26297565	7.21732157
ESR1	0.86045578	13.7931035
MMP11	0.73539923	16.1637931
NAT1	0.62401171	16.1637931
MIA	0.6210077	16.1637931
BAG1	0.49898233	18.3673469
KRT17	0.3191269	18.3673469
GPR160	0.18611141	18.3673469
PGR	0.13378854	18.3673469
MYC	-0.1918202	7.95755968
CXXC5	-0.4822267	7.95755968
ACTR3B	-0.569305	7.95755968
PHGDH	-0.6870804	7.95755968
TMEM45B	-0.8212657	7.95755968
PTTG1	-0.836089	7.95755968
BCL2	-0.9263621	7.95755968
SLC39A6	-1.0662884	4.31034483
BLVRA	-1.127455	3.54679803
BIRC5	-1.166172	3.54679803
CENPF	-1.3409063	1.88087774
UBE2C	-1.4564618	1.88087774
MKI67	-1.6871412	1.14942529
EXO1	-1.9595977	1.14942529
CCNB1	-2.2910507	0
CDC20	-2.3393368	0
UBE2T	-2.3679123	0
RRM2	-2.5099799	0
CDC6	-2.5760795	0
CCNE1	-2.6132856	0
CDCA1	-2.660327	0
ANLN	-2.7131414	0
CDH3	-2.9998097	0
KIF2C	-3.1565632	0
CEP55	-3.3461766	0
MYBL2	-3.405738	0
ORC6L	-4.2898388	0
MELK	-4.3151181	0
TYMS	-4.3920362	0
KNTC2	-7.0013982	0
