# Supplementary material for: Phenotypic changes of HER2-positive breast cancer during and after dual HER2 blockade
Source: Nat Commun. 2020 Jan 20;11:385. doi: 10.1038/s41467-019-14111-3 (PMC6971277; doi:10.1038/s41467-019-14111-3)
Supplement: Supplementary file 3 — Reporting Summary [file 41467_2019_14111_MOESM3_ESM.pdf]

nature research

Corresponding author(s): Aleix Prat

Last updated by author(s): Dec 10, 2019

Reporting Summary

Nature Research wishes to improve the reproducibility of the work that we publish. This form provides structure for consistency and transparency in reporting. For further information on Nature Research policies, see Authors & Referees and the Editorial Policy Checklist.

Statistics

For all statistical analyses, confirm that the following items are present in the figure legend, table legend, main text, or Methods section.

n/a	Confirmed	
☐	☒	The exact sample size (n) for each experimental group/condition, given as a discrete number and unit of measurement
☐	☒	A statement on whether measurements were taken from distinct samples or whether the same sample was measured repeatedly
☐	☒	The statistical test(s) used AND whether they are one- or two-sided<br>*Only common tests should be described solely by name; describe more complex techniques in the Methods section.*
☒	☐	A description of all covariates tested
☒	☐	A description of any assumptions or corrections, such as tests of normality and adjustment for multiple comparisons
☐	☒	A full description of the statistical parameters including central tendency (e.g. means) or other basic estimates (e.g. regression coefficient) AND variation (e.g. standard deviation) or associated estimates of uncertainty (e.g. confidence intervals)
☐	☒	For null hypothesis testing, the test statistic (e.g. F, t, r) with confidence intervals, effect sizes, degrees of freedom and P value noted<br>*Give P values as exact values whenever suitable.*
☒	☐	For Bayesian analysis, information on the choice of priors and Markov chain Monte Carlo settings
☒	☐	For hierarchical and complex designs, identification of the appropriate level for tests and full reporting of outcomes
☒	☐	Estimates of effect sizes (e.g. Cohen's d, Pearson's r), indicating how they were calculated

Our web collection on statistics for biologists contains articles on many of the points above.

Software and code

Policy information about availability of computer code

Data collection	No software used for data collection.
Data analysis	R software version 3.4.3.

For manuscripts utilizing custom algorithms or software that are central to the research but not yet described in published literature, software must be made available to editors/reviewers. We strongly encourage code deposition in a community repository (e.g. GitHub). See the Nature Research guidelines for submitting code & software for further information.

Data

Policy information about availability of data

All manuscripts must include a data availability statement. This statement should provide the following information, where applicable:
- Accession codes, unique identifiers, or web links for publicly available datasets
- A list of figures that have associated raw data
- A description of any restrictions on data availability

Gene expression and in vitro data are provided as a Source Data file as indicated in each specific paragraph of the Methods section, and are also available from the corresponding author upon reasonable request.

Field-specific reporting

Please select the one below that is the best fit for your research. If you are not sure, read the appropriate sections before making your selection.

☒ Life sciences ☐ Behavioural & social sciences ☐ Ecological, evolutionary & environmental sciences

Life sciences study design

All studies must disclose on these points even when the disclosure is negative.

Sample size	No sample size was calculated for gene expression analysis of clinical data, as this was a retrospective analysis of clinical trial samples. All samples available were analyzed. In vitro experiments included technical triplicates and were repeated at least three times.
Data exclusions	No data were excluded.
Replication	All in vitro experiments included technical triplicates and were successfully repeated at least three times.
Randomization	For gene expression analysis of clinical data was a retrospective and descriptive analysis of clinical samples. For all in vitro experiments, three technical replicates were used for each experimental group.
Blinding	No blinding included as this was a retrospective and descriptive analysis of clinical samples. No blinding was possible for in vitro experiments.

Reporting for specific materials, systems and methods

We require information from authors about some types of materials, experimental systems and methods used in many studies. Here, indicate whether each material, system or method listed is relevant to your study. If you are not sure if a list item applies to your research, read the appropriate section before selecting a response.

Materials & experimental systems

n/a	Involved in the study	
☐	☒	Antibodies
☐	☒	Eukaryotic cell lines
☒	☐	Palaeontology
☒	☐	Animals and other organisms
☒	☐	Human research participants
☐	☒	Clinical data

Methods

n/a	Involved in the study	
☒	☐	ChIP-seq
☐	☒	Flow cytometry
☒	☐	MRI-based neuroimaging

Antibodies

Antibodies used	HER2 (D8F12) #4290 Lot2 Cell Signaling Technologies<br>Phospho-HER2 (Tyr1221/1222) (6B12) #2243 Lot 12 Cell Signaling Technologies<br>AKT #9272 Lot 18 Cell Signaling Technologies<br>Phospho-Akt (Ser473) (D9E) #4060 Lot 5 Cell Signaling Technologies<br>Phospho-RB (Ser807/811) (D20B12) #8516 Lot 6 Cell Signaling Technologies<br>Cyclin D1 (92G2) #2978 Lot 13 Cell Signaling Technologies<br>GAPDH (14C10) #2118 Lot 6 Cell Signaling Technologies<br>IRDye 800CW Donkey anti-Rabbit IgG. #926-32213. LI-COR Biosciences
Validation	All antibodies used were previously validated as noted by Cell Signaling Technologies in each antibody datasheet

Eukaryotic cell lines

Policy information about cell lines

Cell line source(s)	BT474, SKBR3 , MCF7 , HCC1954 were obtained from the American Type Culture Collection (ATCC)
Authentication	All cell lines were authenticated using Human 9-Marker STR Profile and Interspecies Contamination Test by IDEXX BioAnalytics
Mycoplasma contamination	All cell lines tested negative for mycoplasma contamination
Commonly misidentified lines<br>(See ICLAC register)	*Name any commonly misidentified cell lines used in the study and provide a rationale for their use.*

Clinical data

Policy information about clinical studies

All manuscripts should comply with the ICMJE guidelines for publication of clinical research and a completed CONSORT checklist must be included with all submissions.

Clinical trial registration	The clinical data used in this study were obtained from the PAMELA clinical trial: NCT01973660
Study protocol	http://www.gruposolti.org/documents/20182/39281/PAMELA+protocolo_v4+0-Enmienda2_SCC_EN.pdf/177a192e-cd0f-44e2-9fdc-9db1a41becb6
Data collection	The clinical data used in this study were obtained from the PAMELA clinical trial
Outcomes	This was a retrospective and exploratory analysis of gene expression data from the PAMELA clinical trial

Flow Cytometry

Plots

Confirm that:

- [x] The axis labels state the marker and fluorochrome used (e.g. CD4-FITC).
- [x] The axis scales are clearly visible. Include numbers along axes only for bottom left plot of group (a 'group' is an analysis of identical markers).
- [ ] All plots are contour plots with outliers or pseudocolor plots.
- [x] A numerical value for number of cells or percentage (with statistics) is provided.

Methodology

Sample preparation	Breast cancer cell lines were fixed in 70% cold ethanol. Propidium Iodide (PI) was used to stain total DNA.
Instrument	BD FacsDiva analyzer
Software	BD FacsDiva analyzer
Cell population abundance	no post-sorting
Gating strategy	Singlets

- [ ] Tick this box to confirm that a figure exemplifying the gating strategy is provided in the Supplementary Information.
